# Correction to Heat inactivation of foetal bovine serum performed after EV‐depletion influences the proteome of cell‐derived extracellular vesicles

**DOI:** 10.1002/jev2.12411

**Published:** 2024-01-31

Urzì, O., Bergqvist, M., Lässer, C., Moschetti, M., Johansson, J., D′Arrigo, D., Olofsson Bagge, R., & Crescitelli, R. (2024). Heat inactivation of foetal bovine serum performed after EV‐depletion influences the proteome of cell‐derived extracellular vesicles. *Journal of Extracellular Vesicles*, 13, e12408. https://doi.org/10.1002/jev2.12408

In the originally published article, a portion of Figure 3 was mislabeled. The section labeled “MML1” should be labeled “Medium + FBS”. The corrected figure is shown below. This has been corrected in the online version of the article.

**FIGURE 3:**
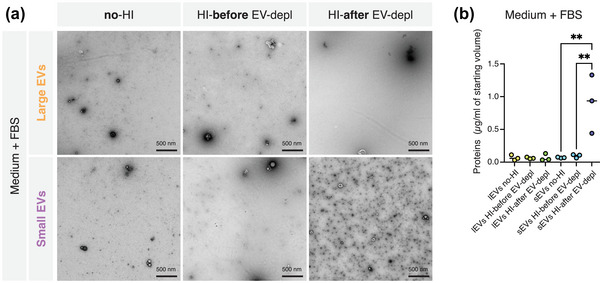


We apologize for this error.

